# Cloning, Characterization, and Expression Analysis of Three *FAD8* Genes Encoding a Fatty Acid Desaturase from Seeds of *Paeonia ostii*

**DOI:** 10.3390/molecules23040929

**Published:** 2018-04-17

**Authors:** Jing Sun, Ming Chen, Mengyuan Zhu, Yu Jiang, Jiasong Meng, Daqiu Zhao, Jun Tao

**Affiliations:** Jiangsu Key Laboratory of Crop Genetics and Physiology, College of Horticulture and Plant Protection, Yangzhou University, Yangzhou 225009, China; jingsun@yzu.edu.cn (J.S.); chenmingyangda@163.com (M.C.); mengyuanzhu199301@163.com (M.Z.); jiangyuyangda@163.com (Y.J.); jsmeng@yzu.edu.cn (J.M.); dqzhao@yzu.edu.cn (D.Z.)

**Keywords:** *Paeonia ostii*, *FAD8*, bioinformatics, quantitative real-time PCR, subcellular localization

## Abstract

The *FAD8* gene catalyzes the conversion of diene fatty acids to triene fatty acids and is a key enzyme that determines the synthesis of alpha-linolenic acid. In this study, the full-length cDNAs of *FAD8-1*, *FAD8-2*, and *FAD8-3* are cloned from *Paeonia ostii* T. Hong & J. X. Zhang and named as *PoFAD8-1*, *PoFAD8-2*, and *PoFAD8-3*. Their open reading frame is 1203 bp, 1152 bp, and 1353 bp which encoded 400, 371, and 450 amino acids. The molecular weights of the amino acids are 46 kDa, 43 kDa, and 51 kDa while the isoelectric points are 7.34, 8.74, and 9.23, respectively. Bioinformatics analysis shows that all three genes are hydrophobic-hydrophobic, *PoFAD8-1* has three transmembrane domains, and *PoFAD8-2* and *PoFAD8-3* have two transmembrane domains. Multiple series alignment and phylogenetic analysis revealed that *PoFAD8-1* and *PoFAD8-2* are closely related while *PoFAD8-3* is more closely related to *Paeonia delavayi*. Subcellular localization results showed that *PoFAD8-1* was located on the ER membrane and *PoFAD8-2* and *PoFAD8-3* were located on the chloroplast membrane. The relative expression level of *PoFAD8-1* in seeds is very high. *PoFAD8-2* expressed more in the ovary than the other two genes. *PoFAD8-3* was highly expressed in roots, stems, leaves, petals, and ovaries.

## 1. Introduction

Fatty acid desaturase (*FAD*) is a key enzyme in plant lipid metabolism, which can promote the conversion of fatty acids to unsaturated fatty acids. At present, many genes involved in the process of fatty acid desaturase metabolism have been isolated from many plants. In model plant *Arabidopsis thaliana*, they are *FAD2*, *FAD3*, *FAD6*, *FAD7*, and *FAD8* [1]. Since it is responsible for the catalytic material, it will be divided into two major categories of ω-3 and ω-6, which entails catalytic unsaturated fatty acid synthesis of fatty acid dehydrogenase [2,3]. The present studies show that *FAD7* and *FAD8* are all expressed in plastids, which mainly affect the composition of the thylakoid membrane lipids. So far, there are many studies on the function of *FAD3* and *FAD7* genes, but little research on the *FAD8* gene [4,5,6]. The *FAD8* gene was first found in *Arabidopsis thaliana* in 1994. It is located in the chloroplast of plant cells and uses ferredoxin as an electron donor to introduce a double bond at the omega-3 fatty acid position, which catalyzed the conversion of diene fatty acids (linoleic acid) to triene fatty acids (linolenic acid) [7]. *FAD8* is also a key enzyme that determines the synthesis of alpha-linolenic acid (ALA) [8]. In *Arabidopsis thaliana*, *FAD8* gene has been found to be constantly expressed at room temperature and its transcriptional regulation is easily induced by low temperatures [9]. Moreover, the physiological process of converting linoleic acid into linolenic acid can increase the degree of unsaturation of cell membrane lipids and it is one of the main ways for plants to adapt to low-temperature environments [10]. In addition to its association with low temperatures, *FAD8* is also involved in the defense response mediated by jasmonic acid [11], ABA (abscisic acid), and SA (salicylic acid) [12]. *FAD8* in tobacco also exhibits the anti-drought stress function [13] and corn *FAD8* shows salt stress resistance [14].

*Paeonia ostii* T. Hong & J. X. Zhang, also known as Tongling peony, is mainly produced in Tongling County in the Anhui Province of China. *Paeonia ostii* T. Hong & J. X. Zhang belongs to *Paeoniaceae* and is the main species of peony oil. Peony seeds are wrapped in a star-shaped pod, which contains large amounts of unsaturated fatty acids (UFAs > 90%) and a high proportion of omega-3 fatty acids. These substances are associated with the prevention of various diseases such as cancer, cardiovascular disease, inflammation, and autoimmune diseases [15,16]. Studies have shown that *FAD2* and *FAD8* are highly expressed in peony seeds, which indicates that *FAD2* and *FAD8* may play a crucial role in forming polyunsaturated fatty acids. Among them, the relative expression level of *FAD8* is much higher than *FAD2* [17]. Li’s research shows that the high content of ALA in peony seeds is due to the activity and abundance of the *FAD8* enzyme [17]. Therefore, as a new type of oilseed crop, it is necessary to study the expression pattern of the *FAD8* gene in peony plants. At present, there are few reports on the expression pattern and function of the *PoFAD8* gene in *Paeonia ostii*. We obtained three *PoFAD8* genes by cloning from *Paeonia ostii* T. Hong & J. X. Zhang to explore the role of *PoFAD8-1*, *PoFAD8-2*, and *PoFAD8-3*.

## 2. Materials and Methods

### 2.1. Plant Material and Treatments

The experiment was conducted with the white peony cultivar *Paeonia ostii* T. Hong & J. X. Zhang planted in the Peony Germplasm Resources Park (32°23′ N, 119°24′ E) of Yangzhou University in Yangzhou, Jiangsu Province. The roots, stems, leaves, petals, ovaries, and seeds of 55 days, 70 days, 85 days, and 100 days after flowering were collected. At least three plant samples are repeated three times for each experiment. All samples were collected and placed in liquid nitrogen immediately for rapid freezing and then placed in a −80 °C cryogenic refrigerator to save them. 

### 2.2. Extraction of Seed Oil and Measurement by GC-MS

The dried seed samples of different stages were peeled and crushed into powdered form. The oil content of developing seeds was extracted as described in our previous study [18]. Oil was extracted through an ultrasonic assisted extraction method. 30.0 g of seed powder was weighed and transferred into a 500 mL round bottom flask. Then n-hexane was mixed in with the material at a 1:13 ratio (g/mL). Afterwards, the flask was connected to a condenser tube and placed in the ultrasonic cleaning machine to perform an oil extraction at 70 °C and 200 W of power at a fixed frequency of 80 kHz. After extracting for 1.5 h, the mixture was centrifuged at 4000 r/min for 10 min. The supernatant was separated and dried by rotary evaporation at 50 °C for oil separation and n-hexane recovery. 

The fatty acids (FAs) of *Paeonia ostii* seeds were extracted and were methylated according to procedures in the previous study [19]. FA content was analyzed through a gas chromatograph-mass (GC-MS) spectrometer (GC7890A/MS5975C, Agilent Technologies, Santa Clara, CA, USA) with HP-88 column (100 m × 0.25 mm i.d, 0.20-μm film thickness; Agilent). An internal standard curve was used to calculate unsaturated fatty acid content. The methyl heptadecanoate was used as the internal standard and the fatty acid methyl esters (FAMEs) were measured in each sample. The FAMEs of samples were recorded as milligrams per gram of dry weight (DW). All samples were analyzed in triplicate.

### 2.3. Cloning of Full-Length PoFAD8-1, PoFAD8-2, and PoFAD8-3 cDNA 

The instructions of the TaKaRa MiniBEST Plant RNA Extraction Kit were used to extract the total RNA of the seeds and leaves. After RNA extraction, 1 μL was taken for agarose gel electrophoresis to measure its quality and concentration while the rest was stored at −80 °C in a refrigerator.

Using the designed primers to amplify the cDNAs of *PoFAD8-1*, *PoFAD8-2*, and *PoFAD8-3*, the amplified PCR products were detected by using 1% agarose gel electrophoresis. The target band was recovered by TaKaRa’s MiniBEST Agarose Gel cDNA Extraction Kit and then TA cloned using the pEASYTM-T5 Zero Cloning Kit. Then, the cloning vector was transformed into *E. coli DH5α* competent cells. Then after antibiotic (ampicillin) screening, the positive clones were finally sent to Shanghai Bioengineering Co., Ltd. (Shanghai, China) for sequencing.

### 2.4. Bioinformatic Analysis 

The full-length open reading frame of cDNA and the encoded amino acids were predicted using Bioedit software. Biological software DNAMAN 5.0 was used to analyze translational sequences. The homology alignment of the NCBI Blast was carried out using the on-line tool NCBI Blast and the phylogenetic tree was constructed by using the NJ (Neighbor-joining) adjoining method of MEGA5.0 with the current major oil plants. The softwares Prot Param (http://web.expasy.org/protparam/), Hydrophobicity ProtScale (http://web.expasy.org/cgi-bin/protscale/protscale.pl), TMHMM2.0 (http://www.cbs.dtu.dk/services/TMHMM-2.0/), SMART (http://smart.embl-heidelberg.de/smart/), and SOPMA (https://npsa-prabi.ibcp.fr/cgi-bin/npsa_automat.pl?page=/NPSA/npsa_sopma.html) were used to analyze the physicochemical properties, hydrophilicity, and hydrophobicity as well as the transmembrane domains and secondary structure of the gene encoding proteins, respectively.

### 2.5. Protein Subcellular Localization 

To further confirm the plastidial localization of the *PoFAD8-1*, *PoFAD8-2*, and *PoFAD8-3* protein, full-length ORF PCR production of target genes that contain the *Bsa*I/*Eco31*I restriction cut sites were cut by the endonuclease (Takara, Tokyo, Japan). Purified fragments were then ligated into the vector pBWA(V)HS-ccdb-GLosgfp. The pBWA(V)HS-ccdb-GLosgfp vector contains the Cauliflower mosaic virus (CaMV) 35S promoters and the mGFP reporter gene was fused with *PoFAD8-1*, *PoFAD8-2*, and *PoFAD8-3.* The p35S::*PoFAD8-1*-GFP, p35S::*PoFAD8-2*-GFP, and p35S::*PoFAD8-3*-GFPconstructs along with the empty pBWA(V)HS-GFP, vector pBWA(V)HS-ER-mKate vector (endoplasmic reticulum positioning signal), and pBWA(V)HS-Chloroplast-mKate vector (chloroplast positioning signal) were transformed into *O. sativa* protoplasts using a modified procedure described previously [20]. 

The rice seedlings were dark-cultured at 25 °C for 1 to 2 weeks. Fresh rice seedlings were cut into sections and added with the appropriate amount of enzyme solution (1.5% Cellulase R10, 0.75% Macerozyme R10, 0.6 M mannitol, 10 mM MES pH 5.7, 10 mM CaCl_2_, and 0.1% BSA). Gently shake (20–30 rpm) for 3 h at 15–20 °C. The collected protoplasts were transformed under PEG mixture [40% (w/v) PEG 4000, 2 M mannitol, and 0.1 M CaCl2] with 10 µg target plasmid. After incubate at room temperature for 16–24 h, the expression of plasmid was observed under a laser scanning confocal microscope.

### 2.6. Gene Expression Analysis

Using Bio-Rad’s CFX96 ReakTime System (Bio-Rad, Hercules, CA, USA), the gene sequences of *PoFAD8-1*, *PoFAD8-2*, and *PoFAD8-3* were analyzed by qRT-PCR (real-time quantitative PCR) in the roots, stems, leaves, petals, ovary, and seed (60 d) of all parts of the seedlings and seeds of the 55 d, 70 d, 85 d, and 100 d expression differences. Real-time quantitative PCR was performed using the PrimeScript RT reagent Kit with gDNA Eraser kit (TaKaRa, Tokyo, Japan). The internal reference for the *Paeonia ostii*’s ubiquitin gene with specific expression primers shown in Table 1, have amplification conditions as follows: denaturation at 94 °C for 3 min, denaturation at 94 °C for 30 s, annealing at 54 °C for 30 s, extension at 72 °C for 1 min, 35 cycles, and extension at 72 °C for 10 min. The reaction Ct values were collected using Bio-Rad CFX Manager V1.6.541.1028 software. Repeat three times for each sample. The final result was calculated using the 2^−ΔΔ^*^C^*^t^ method [21]. 

### 2.7. In Vitro Expression of FAD8-1 through Cell-Free Expression System

The expression plasmid of *PoFAD8-1* was constructed. The full-length ORF of *PoFAD8-1* was amplified and connected onto pET21a vector, which was promoted by the T7 promoter and labeled with a 10 xhis tag. Additionally, 30 μL mixture contained *E. coli* extracts, feed buffers, amino acids, and T7 RNA polymerase from the Expressway™ Mini Cell-Free Expression System (Thermo Fisher, Waltham, MA, USA) and 20 μL plasmid pET21a-*PoFAD8-1* (concentration at 150 μg/mL) was added into the dialysis box. Then the cassette was immersed in the feed buffer at 30 °C overnight. The reaction solution was taken and centrifuged at 12,000 rpm for 10 min at 4 °C. The supernatant was discarded and the precipitates were diluted and detected by SDS-PAGE. 

## 3. Results

### 3.1. Cloning and Nucleotide Sequence Analysis of PoFAD8-1, PoFAD8-2, and PoFAD8-3

The cDNA fragments of *PoFAD8-1*, *PoFAD8-2*, and *PoFAD8-3* were obtained by PCR amplification from *Paeonia ostii* T. Hong & J. X. Zhang. After sequencing the PCR products, their sequences were contrasted with *FAD* genes of *Arabidopsis thaliana* and blasted at Tair (http://www.arabidopsis.org/). Afterward, they were identified as *PoFAD8-1*, *PoFAD8-2*, and *PoFAD8-3*. Their sequences were uploaded onto NCBI and their accession numbers were MH049427, MH049428, and MH049429, respectively. Sequence analysis showed that the ORF of the *PoFAD8-1* gene was 1203 bp in length and encoded 400 amino acids with a molecular weight of 46 kDa and an isoelectric point of 7.34. The ORF of the *PoFAD8-2* gene was 1152 bp in length and encoded 371 amino acids. The molecular weight of the gene was 43 kDa and the isoelectric point was 8.74. The ORF of *PoFAD8-3* gene is 1353 bp in length and encodes a protein of 450 amino acids with a molecular weight of 51 kDa and an isoelectric point of 9.23. Genomic DNA was amplified by PCR using primers *PoFAD8-1*, *PoFAD8-2*, and *PoFAD8-3*.

**Figure 1:**
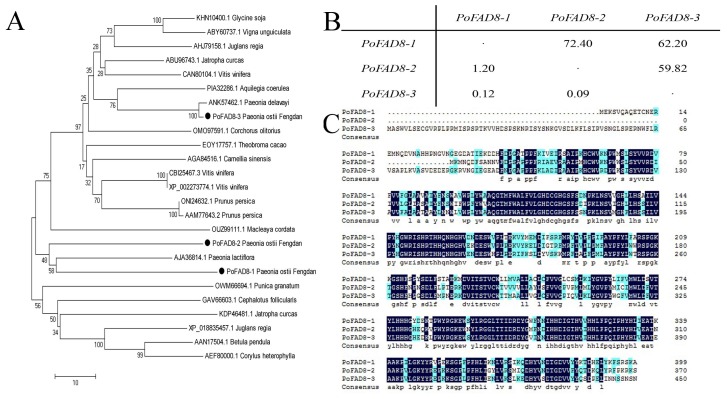
Multi-series alignment and phylogenetic analysis. (**A**) Neighbor-Joining System evolutionary tree of *PoFAD8-1*, *PoFAD8-2*, and *PoFAD8-3* genes with another 17 species. The GenBank accession numbers are: *Glycine soja* (*GsFAD8*, KHN10400.1), *Vigna unguiculata* (*VuFAD8*, ABY60737.1), *Juglans regia* (*JrFAD8*, AHJ79158.1), *Jatropha curcas* (*JcFAD8*, ABU96743.1) *Vitis vinifera* (*VvFAD8*, CAN80104.1), *Aquilegia coerulea* (*AcFAD8*, PIA32286.1), *Paeonia delavayi* (*PdFAD8*, ANK57462.1), *Corchorus olitorius* (*CoFAD8*, OMO97591.1), *Theobroma cacao* (*TcFAD8*, EOY17757.1), *Camellia sinensis* (*CsFAD8*, AGA84516.1), *Vitis vinifera* (*VvFAD8*, CBI25467.3), *Vitis vinifera* (*VvFAD8*, XP_002273774.1), *Prunus persica* (*PpFAD8*, ONI24632.1), *Prunus persica* (*PpFAD8*, AAM77643.2), *Macleaya cordata* (*McFAD8*, OUZ99111.1), *Paeonia lactiflora* (*PlFAD8*, AJA36814.1), *Punica granatum* (*PgFAD8*, OWM66694.1), *Cephalotus follicularis* (*CfFAD8*, GAV66603.1), *Jatropha curcas* (*JcFAD8*, KDP46481.1), *Juglans regia* (*JrFAD8*, XP_018835457.1), *Betula pendula* (*BpFAD8*, AAN17504.1), and *Corylus heterophylla* (*ChFAD8*, AEF80000.1). The horizontal scale shows the difference number per 100 residues from the Clustal W alignment. (**B**) Comparisons of *PoFAD8* paralogues. Ka/Ks and amino acid sequence identity (%) values for pairwise comparisons of *PoFAD8* paralogues are shown. Ka/Ks values are shown below the diagonal while amino acid sequence identity (%) values are shown above the diagonal. (**C**) Protein sequence alignment of *PoFAD8-1*, *PoFAD8-2*, and *PoFAD8-3* genes. The blue black and light blue boxes show the same and similar amino acids, respectively.

To compare the differences of the sequence of *PoFAD8-1*, *PoFAD8-2*, and *PoFAD8-3* (Supplementary Figure S1), DNAMAN software was used to compare the amino acid composition and the number of *PoFAD8-1*, *PoFAD8-2*, and *PoFAD8-3* genes with the protein sequence, which is shown in Table 2 and Figure 1C. MEGA software was used to compare the amino acid sequences of *PoFAD8-1*, *PoFAD8-2*, and *PoFAD8-3* with the *FAD8* gene of 17 species. The Neighbor-Joining system is constructed to set the number of bootstrap to be 1000 and the results are shown in Figure 1A. Cluster analysis showed that *PoFAD8-1* was closely related to *PoFAD8-2*. *PoFAD8-3* is more closely related to *Paeonia delavayi*. *PoFAD8-1* and *PoFAD8-2* shared a close evolutionary relationship with *Paeonia lactiflora* and *PoFAD8-3* shared close evolutionary origins with *Aquilegia coerulea*. The *PoFAD8-1* and *PoFAD8-2* genes are clustered in different regions with *PoFAD8-3*, which indicates that the *PoFAD8-1* and *PoFAD8-2* genes are more distantly related to *PoFAD8-3*. DNAMAN was used to do the pairwise alignment of three genes. The pairwise comparison of *PoFAD8-1*, *PoFAD8-2*, and *PoFAD8-3* with MEGA for nucleic acid molecular evolution analysis show that *PoFAD8-1* and *PoFAD8-2* genes have high identity and their identity rate with *PoFAD8-3* is low. Ka/Ks values of *PoFAD8-1* and *PoFAD8-2* are close to 1, which indicates that these two genes have not received or seldom been under the pressure of natural selection and they are moderately evolved. The Ka/Ks values of *PoFAD8-1* and *PoFAD8-3* as well as *PoFAD8-2* and *PoFAD8-3* are far less than 1. This can be explained by the gene being subject to purification. The results are shown in Figure 1B.

### 3.2. Bioinformatic Analysis of the PoFAD8-1, PoFAD8-2, and PoFAD8-3 Protein

**Figure 2:**
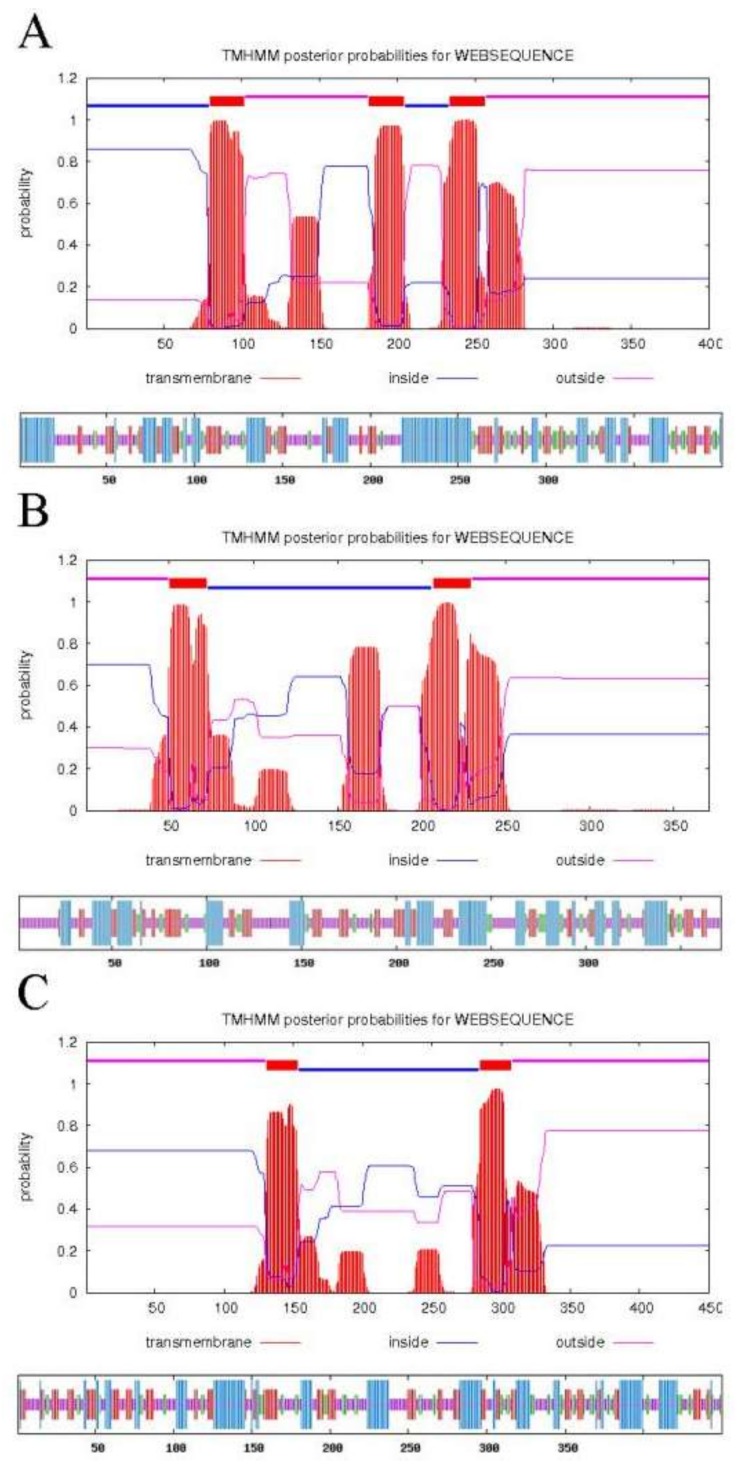
Transmembrane domain prediction and secondary structure prediction of *PoFAD8-1*, *PoFAD8-2*, and *PoFAD8-3*. (**A**) Transmembrane domain prediction and secondary structure prediction of *PoFAD8-1*. (**B**) Transmembrane domain prediction and secondary structure prediction of *PoFAD8-2*. (**C**) Transmembrane domain prediction and secondary structure prediction of *PoFAD8-3*.

The analysis results are shown in Figure 2 below. It can be seen from Figure 2A that *PoFAD8-1* has three predicted transmembrane domains of 80 to 102, 182 to 204, and 234 to 256 amino acids. The protein encoded by *PoFAD8-1* has a minimum hydrophobicity of −2.867 and a maximum of 3.267, which indicates that the protein is a hydrophobic protein. In the secondary structure prediction of *PoFAD8-1*, the total alpha helix has 140 amino acids, which accounts for 35%. The total extended strand is 80 amino acids, which accounts for 20%. The total random coil is 131 amino acids, which accounts for 32.75%. The total beta turn is 49 amino acids, which accounts for 12.25%. The alpha helix, extended strand, random coil, and beta turn run through the entire amino acid chain. In Figure 2B, *PoFDA8-2* has two transmembrane domains of 50 to 72 and 207 to 229 amino acids. In addition, the protein encoded by the *PoFAD8-2* had a minimum hydrophobicity of −2.867 and a maximum of 3.433, which indicates that the protein is a hydrophobic protein. The secondary structure of *PoFAD8-2* predicted that the total alpha helix has 105 amino acids, which accounts for 28.3%. The total extended strand has 79 amino acids, which accounts for 21.29%. The total random coil has 150 amino acids, which accounts for 40.43%. The total beta turn has 37 amino acids, which accounts for 9.97%. The alpha helix, extended strand, random coil, and beta turn run through the entire amino acid chain. In Figure 2C, *PoFAD8-3* has two transmembrane domains of amino acids 131 to 153 and 285 to 307, respectively. The protein encoded by the *PoFAD8-3* gene has a minimum hydrophobicity of −2.867 and a maximum of 3.211, which indicates that the protein is a hydrophobic protein. In the secondary structure prediction of *PoFAD8-3*, the total alpha helix has 126 amino acids, which accounts for 28%. The total extended strand has 101 amino acids, which accounting for 22.44%, and the total random coil has 163 amino acids accounting for 36.22%. Additionally, the total beta turn has 60 amino acids, which accounts for 13.33%. The alpha helix, extended strand, random coil, and beta turn run through the entire amino acid chain.

### 3.3. Subcellular Localization of the PoFAD8-1, PoFAD8-2, and PoFAD8-3 Protein

To determine the subcellular localization of the *PoFAD8-1*, *PoFAD8-2,* and *PoFAD8-3* protein, the fusion protein expression vector pBWA(V)HS-*FAD8-1*-GFP, pBWA(V)HS-*FAD8-2*-GFP, and pBWA(V)HS-*FAD8-3*-GFP were transformed into the protoplast of rice by the PEG method using an empty vector pBWA(V)HS-GFP as a control. The pBWA(V)HS-ER-mKate vector (endoplasmic reticulum positioning signal) and vector pBWA(V)HS-Chloroplast-mKate vector (chloroplast positioning signal) were used as a subcellular co-localization vector.

**Figure 3:**
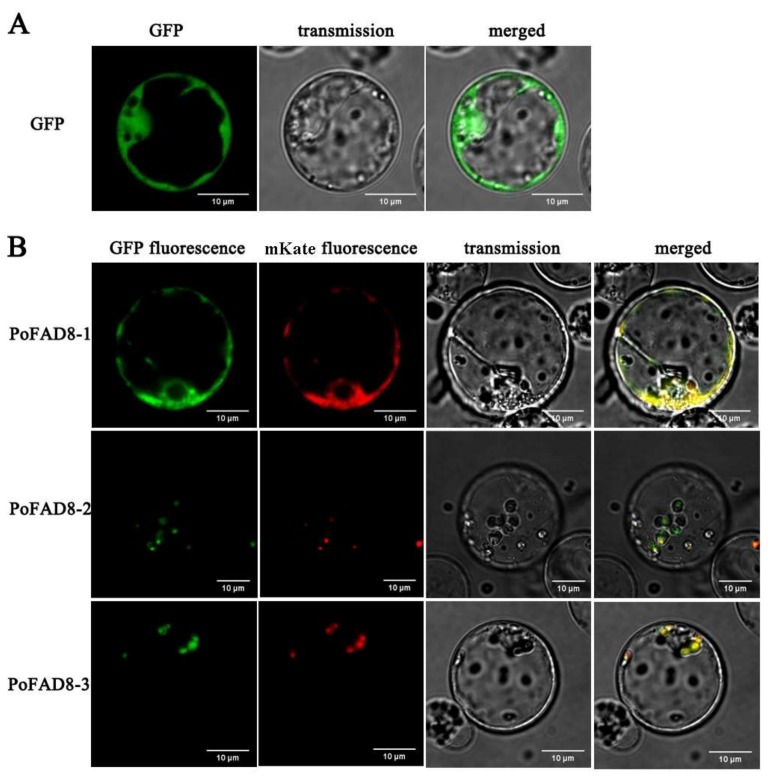
Subcellular localization of *PoFAD8-1*, *PoFAD8-2*, and *PoFAD8-3* in rice protoplasts.

Using the GFP empty vector as a positive control, transgenic rice protoplasts were stimulated by 488 nm to emit green fluorescence. As shown in Figure 3A, the green fluorescence sites were widely distributed in the whole plasma membrane system and nucleus but not in the vacuole (central dark part) and chloroplast. Figure 3B showed that *PoFAD8-1* only appeared on the endoplasmic reticulum membrane and was consistent with the localization of mKate. This result indicated that *PoFAD8-1* was located on the endoplasmic reticulum membrane. The fluorescence intensity of the *PoFAD8-1*-GFP vector (B) was less than the empty vector control. However, the green fluorescence of the 35S::*FAD8-2*-GFP and 35S::*FAD8-3*-GFP chimeras were located at the chloroplast membrane, which was co-localized with chloroplast-mKate in the protoplasts. These results indicated that *PoFAD8-1* was an ER membrane protein, which was consistent with subcellular localization prediction results and was different from the other two family members *PoFAD8-2* and *PoFAD8-3*.

### 3.4. Expression Pattern of the PoFAD8-1, PoFAD8-2, and PoFAD8-3 Genes

**Figure 4:**
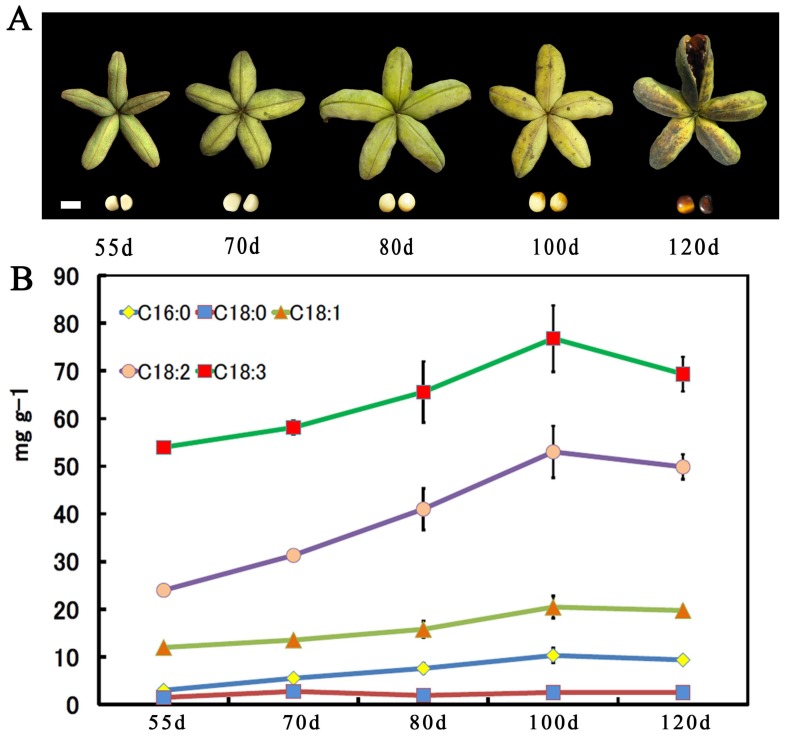
Observation and measurement of lipids across the developmental period of *Paeonia ostii* seeds. (**A**) The developmental progress of *Paeonia ostii* seeds. The pods were harvested at 55 days after pollination (DAP, immature stage) and every approximately 15 days thereafter until 120 DAP (pods containing mature seeds), bar = 1 cm. (**B**) The total fatty acid content at five time points during *Paeonia ostii* seed development (mean ± SD, *n* = 3).

The seed development process was observed from 55 days after pollination until 120 days maturation. The pods of *Paeonia ostii* were hand-collected at approximately fifteen-day intervals from the 55 days after pollination DAP until full maturity, which covers a total range of 65 days including 55 days, 70 days, 85 days, 100 days, and 120 days (a total of five time points). Figure 4A shows that the size and color varied dramatically at different stages of seed development. The fatty acid contents of seeds from five developmental stages of *Paeonia ostii* were characterized by GC–MS and the FA content is depicted in Figure 4B. The results showed that five dominant components were found. These are called α-linolenic acid (C18:3Δ9c, 12c, 15c), linoleic acid (C18:2Δ9c, 12c), oleic acid (C18:1Δ9c), stearic acid (C18:0), and palmitic acid (C16:0). The combined content of these five FAs was more than 99.3% of total FAs at 100 days and it was always predominant across the seed developmental stages. The high proportion of n-3 FAs is quite rare in oil crops. Other minor FAs (<1.0%) were also detected including myristic acid (C14:0), palmitoleic acid (C16:1Δ9c), cis-11-octadecenoic acid (C18:1Δ11c), eicosanoic acid (C20:0), and cis-11-eicosenoic acid (C20:1Δ11c).

**Figure 5:**
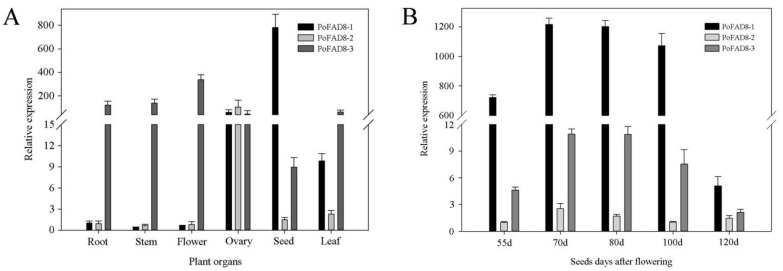
*PoFAD8-1*, *PoFAD8-2*, and *PoFAD8-3* genes expression were measured during seed development and different organs in the *Paeonia ostii* T. Hong & J. X. Zhang. (**A**) Tissue specific expression of *PoFAD8-1*, *PoFAD8-2*, and *PoFAD8-3*. (**B**) *PoFAD8-1*, *PoFAD8-2*, and *PoFAD8-3* expression during *Paeonia ostii* seed development. The error bar represents the standard deviation of three replicates. The tree peony UBQ was used as an internal control.

We measured the expression of *PoFAD8-1*, *PoFAD8-2*, and *PoFAD8-3* in the roots, stems, leaves, petals, ovaries, and seeds of the genus *Paeonia ostii* T. Hong & J. X. Zhang by qRT-PCR. From Figure 5A, we can see that the expression of *PoFAD8-1* in the ovary and seed is extremely high. The relative expression level of *PoFAD8-1* in seeds was 781, which was 531 and 87 times of that of *PoFAD8-2* and *PoFAD8-3*, respectively. *PoFAD8-1* also has the highest expression among three genes in the measured six organs. *PoFAD8-2* was expressed higher in the ovary than in the other two genes and its relative expression was 105. The expression of *PoFAD8-3* in roots, stems, leaves, petals, and ovaries was high. Among them, the expression of *PoFAD8-3* was far ahead of the other two genes in roots, stems, leaves, and petals and the highest relative expression level in flowers was 338, which were 553 and 427 fold of *PoFAD8-1* and *PoFAD8-2*, respectively. Its expression in the seeds is higher than *PoFAD8-2*.

From Figure 5B, we can see that during the five stages of seed development, *PoFAD8-1* is always expressed the most and is followed by *PoFAD8-3* and *PoFAD8-2*. The expression of *PoFAD8-1* in the first four periods has been much higher than that of *PoFAD8-2* and *PoFAD8-3*. The fifth period was slightly higher than *PoFAD8-2* and *PoFAD8-3*. The trends of the five developmental stages of the three genes were similar. The 55 days, 70 days, and 80 days during these three periods, the expression of three genes increased. Interestingly, the relative expression of *PoFAD8-1* decreased slightly at 100 days. In the 120 days, however, it dropped sharply from 1073 to 5. It is speculated that *PoFAD8-1* may play a major function in ovaries and seeds, which specifically regulate unsaturated fatty acid synthesis. According to the GC-MS measurement of FA content, the highest content of ALA in 85 days may be caused by a high expression of *PoFAD8-1*. The relative expression of *PoFAD8-1* dramatically decreased after 100 days. This is consistent with the decrease of *Paeonia ostii* unsaturated fatty acids after 100 days.

### 3.5. In Vitro Expression of PoFAD8-1 Gene

**Figure 6:**
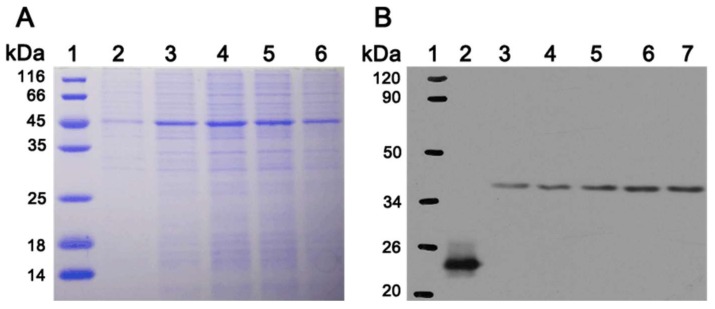
(**A**) The *FAD8-1* protein was synthesized in vitro using the Expressway^TM^ Mini Cell-Free Expression System. Products were loaded onto an SDS-PAGE gel and proteins were then visualized with Coomassie Blue staining. (**B**) Western Blot was also carried out and detected by his antibody. The exogenous positive control protein containing his tag at 23 kDa was used as a control.

Because the subcellular localization of *PoFAD8-1* is different from *PoFAD8-2* and *PoFAD8-3* and its expression level is always t its highest state, it indicated that it may be closely related to the high ALA content of peony seeds. Therefore, the PoFAD8-1 protein was heterologous expressed for further exploring the characteristics. In the earlier period, we used the *Pichia pastoris* expression system, but since *PoFAD8-1* is a multi-spanning membrane protein, the in vitro secretion is very difficult. We did not obtain destination band in SDS-PAGE. After improvement, we constructed the in vitro expression of *PoFAD8-1* driven by using the T7 promoter. We used cell-free expression technology and successfully expressed and purified the *PoFAD8-1* protein. Two-fold dilutions of the cell-free expression system were loaded as 10 μL, 12 μL, 14 μL, 16 μL, and 18 μL (see Figure 6A). After SDS-PAGE and detection, the band size was 45 kDa and was consistent with the prediction. Following a western blot experiment, the results showed that the positive control obtained the correct imprinting of 23 kD and the size of *PoFAD8-1* band was the same as the predicted 45 kD (see Figure 6B). After gel purification and mass spectrometry, the results showed that the amino acid sequence was identical to that of *PoFAD8-1*. It was demonstrated that the *PoFAD8-1* in vitro expression experiment was successful.

## 4. Discussion

At present, there are relatively few studies on the function of *FAD8* genes and they mainly focus on the related gene cloning and expression pattern analysis. Olga PY et al. [22] analyzed the ω-3 fatty acid desaturase gene family of *Gossypium* spp. from the genome level. The transcriptome sequencing revealed that *FAD7*/*8-1* gene could be induced by low temperature. Similarly, a study of the *CsFAD8* gene expression pattern in *Camellia sinensis* showed that the expression of this gene was induced by a low temperature and ABA stress. In addition, functional studies of *Arabidopsis At-FAD8* proved that the gene can regulate the fatty acid content decreased with increasing temperature. However, Susan et al. [7] found that the *FAD8* gene in *Arabidopsis* was induced by cold and expressed in large quantities below 20 °C, which rapidly increased the cold resistance of tissues [23]. Studies on the omega-3 fatty acid dehydrogenase gene in *Glycine max* (Linn.) Merr. showed that hypothermia did not affect the expression levels of *GmFAD8-1* and *GmFAD8-2* [24]. The study of the expression patterns of *CmsFAD2* and *CmsFAD8* genes in both cryopreserved and cold-sensitive *Citrus medica* L. *var. sarcodactylis.* Swingle showed that the expression level of the two genes correlated with the genotypes of the two plants [25,26]. In *Arabidopsis*, two types of omega-3 *FADs* (*FAD3* located in the ER and *FAD7*/*8* located in the chloroplast) catalyze the formation of TA [7,26,27]. Matilda et al. [23] found that, even though the two kinds of ω-3 fatty acid desaturase *FAD7* and *FAD8* in *Arabidopsis* have a high degree of structural homology, the response mechanisms of the two enzymes to the temperature are completely different. They transferred a series of constructed *FAD7-FAD8* fusion genes into *Arabidopsis FAD7-FAD8* double mutants. The effect of each gene on temperature response was examined by Northernm and Southern analysis and fatty acid composition measurements. The results showed that all the transgenic plants transfected into the construct with the *FAD8* C-terminal coding region (44 amino acids) showed a significant decrease in TA levels at high temperature. This is similar to the plants obtained by transgenes with the *FAD8* native structural gene. The expression profile of omega-3 *FAD* gene in *Perilla frutescens* was studied by transcriptome analysis. The results showed that *FAD3* and *FAD7*/*8* were the key genes for ALA synthesis in seeds and leaves, respectively [28]. The expression level of *PfFAD7*/*8* in *Perilla frutescens* leaves was higher than that in seeds, which indicates that they play a preferential role in ALA accumulation in vegetative organs. After heat treatment of *Perilla frutescens* leaves for 48 hours, the transcripts of Shia *ShFAD3-1*/*-2* and *ShFAD7* slightly decreased and the expression of *ShFAD8* slightly increased, which indicates that *ShFAD8* might respond to heat [29].

In *Arabidopsis thaliana*, *FAD7* and *FAD8* are genes with similar functions. However, *FAD8* is highly expressed under low temperature induction [7]. Its function is to catalyze the conversion of plant diene fatty acids (palmitate and linoleic acid) into TAs in the chloroplast. Post-transcriptional regulation of *FAD8* exists and is closely related to the rapid increase of TAs at low temperature and the decrease of TAs at high temperature [23]. As can be seen from the molecular phylogenetic tree, the catalytic TAs-containing fatty acid desaturase gene is a large family of *FAD7*, *FAD8*, and plastid-expressed *FAD3* [30]. In most plants, *FAD7* and *FAD8* have a closer genetic relationship. Both *FAD7* and *FAD8* have similar catalytic functions and different expression and regulation. In phylogenetic tree analysis, the two genes apparently become two branches, but the distances between different plants are larger. In general, the similarity between the two genes *FAD7* and *FAD8* is generally lower than that of *FAD8* among different species.

Peony seed oil contains a lot of unsaturated fatty acids and gradually favored by consumers. The abundant α linolenic acid (C18:3) brings a great nutrition to human health. *FAD*s, known as the fatty acid desaturase, control the key steps of oleic acid transfer to the linoleic acid and α linolenic acid. The regulation mechanism of synthesis of α linolenic acid is still unclear.

Generally *FAD3*, *FAD7*, and *FAD8* are not a saturated fatty acid catalytic enzyme. Plants use them as key enzymes to produce C18:3. *FAD3* is widespread distribution in endoplasm momentum while *FAD7* and *FAD8* distribution on the chloroplast membrane. In this study, the results show that, compared with *FAD8-2* and *FAD8-3, FAD8-1* changed the distribution of the position. However, the subcellular localization experiment proves its location is on the ER instead of the chloroplast membrane. The results suggest that during the evolution of peony, the functional differentiation of *FAD8-1* happened. The subcellular level localization has also changed due to the area being widely distributed in the endoplasmic reticulum in cells. The cell-free protein expression system, also known as the *in vitro* translation system, simulates the life phenomena of biological cells and reproduces the translation and translation process of intracellular proteins. The cell-free protein synthesis system uses an external target mRNA or DNA as a template and artificially supplements the substrates required for protein synthesis and transcription and translation-related factors to achieve the *in vitro* synthesis of the target protein. *PoFAD8-1* is used as a catalytic enzyme that is embedded in the membrane of the endoplasmic reticulum. After purification in vitro, how to make it form the correct spatial conformation and how to perform certain enzyme activities, the laboratory still needs further study.

## 5. Conclusions

In conclusion, we cloned the fatty acid desaturase genes *PoFAD8-1*, *PoFAD8-2*, and *PoFAD8-3* of *Paeonia ostii* T. Hong & J. X. Zhang. A subcellular localization analysis confirmed that PoFAD8-1 is located on the ER membrane while PoFAD8-2 and PoFAD8-3 are located on the chloroplast membrane. The relative expression level of *PoFAD8-1* in seeds was very high, which is also the highest expression level of the three genes in the tested six loci. *PoFAD8-2* expresses more in the ovary than the other two genes. *PoFAD8-3* is highly expressed in roots, stems, leaves, petals, and ovaries. In the five stages of seed development, the expression levels of *PoFAD8-1* in the first four periods were much higher than those in *PoFAD8-2* and *PoFAD8-3* while the fifth period was slightly higher than *PoFAD8-2* and *PoFAD8-3*. We speculate from these results that *PoFAD8-1* has a very close relationship to high content of ALA in peony seed oil. Further, *PoFAD8-1* in vitro expression experiments using the cell-free expression system were successfully performed and allowed other FAD8 members to be expressed in vitro. Since the endoplasmic reticulum is widely distributed in cells, it can provide a high catalytic efficiency for dehydrogenation of fatty acids. The change of PoFAD8-1 distribution in cells expanded its catalytic range, which made a contribution toward the high content of ALA in peony seeds. We believe that this report will provide a lot of information for further study of the peony *FAD* family and provide reference for the production of high ALA content of germplasm resources in the future.

## Figures and Tables

**Table 1:** Primers and sequences of *PoFAD8-1*, *PoFAD8-2*, and *PoFAD8-3* gene cloning.

Gene	Forward Primer (5′-3′)	Reverse Primer (5′-3′)
Ubiquitin	GACCTATACCAAGCCGAAG	CGTTCCAGCACCACAATC
*qFAD8-1*	ACCTTAGAGGAGGGCTTACGACAAT	CGTAGTGGTCTTGCTTGATGCTCCT
*qFAD8-2*	TAGTGGCATGAAGATGAATCAAGAT	CGCTTGCTATTAGTCCCAGAACCAC
*qFAD8-3*	ATGATAGCCCCTCAAAGAATAGAAT	CGCAACATCCCTCACAACATAGC

**Table 2:** Amino acid composition and number of products encoded by cDNAs of *PoFAD8-1*, *PoFAD8-2*, and *PoFAD8-3* gene.

Amino Acids	*PoFAD8-1*	*PoFAD8-2*	*PoFAD8-3*	Amino Acids	*PoFAD8-1*	*PoFAD8-2*	*PoFAD8-3*
Ala (A)	22	14	24	Leu (L)	30	31	40
Arg (R)	14	16	24	Lys (K)	22	17	24
Asn (N)	15	14	20	Met (M)	11	12	9
Asp (D)	22	19	22	Phe (F)	23	22	20
Cys (C)	7	4	5	Pro (P)	23	28	31
Gln (Q)	7	8	5	Ser (S)	25	21	38
Glu (E)	17	10	18	Thr (T)	18	16	17
Gly (G)	25	23	30	Trp (W)	13	15	16
His (H)	28	24	28	Tyr (Y)	23	21	20
Ile (I)	20	22	20	Val (V)	35	34	39

## Supplementary Materials

The following are available online, Figure S1: Sequence alignment of *PoFAD8-1*, *PoFAD8-2*, and *PoFAD8-3*.
