# Supplementary figures and images for: Cloning, Characterization, and Expression Analysis of Three FAD8 Genes Encoding a Fatty Acid Desaturase from Seeds of Paeonia ostii
Source: Molecules. 2018 Apr 17;23(4):929. doi: 10.3390/molecules23040929 (PMC6017405; doi:10.3390/molecules23040929)

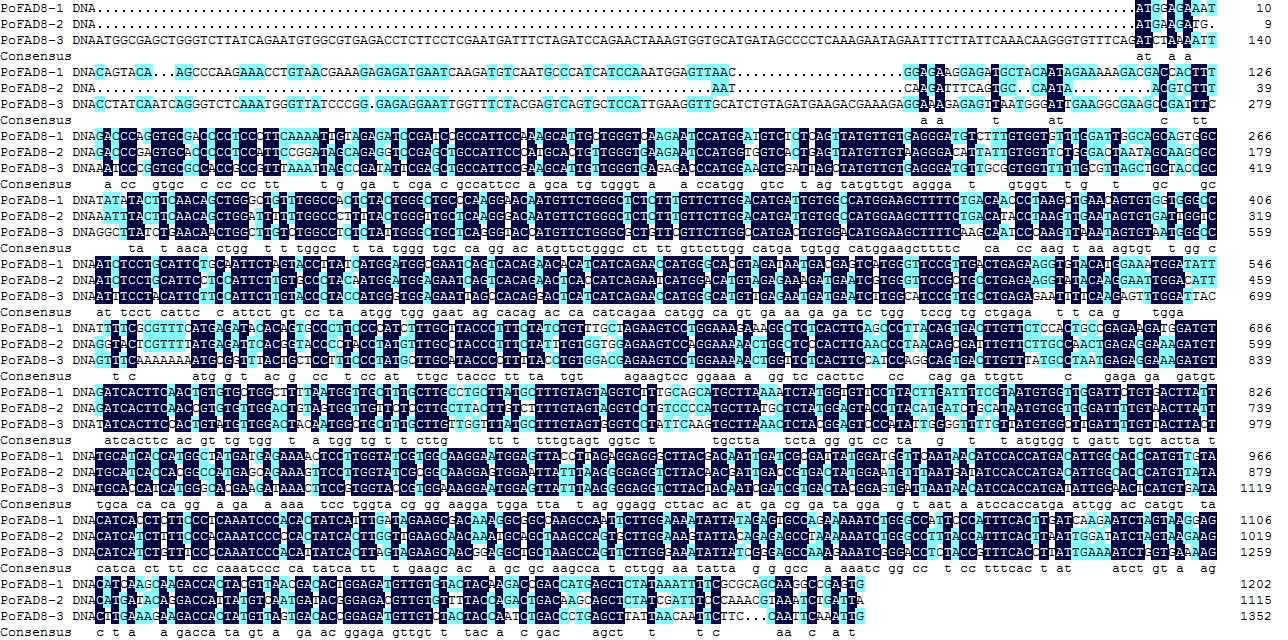

Supplement: Supplementary file 1 [file molecules-23-00929-s001.jpg]
